# The Effect of Multicultural Attitudes and Perceived Intergroup Threat on Attitudes Towards Immigrants in Portugal: A Polynomial Regression With Response Surface Analysis

**DOI:** 10.1177/00332941221149182

**Published:** 2023-01-03

**Authors:** Gabriela Gonçalves, Cátia Sousa, Lily A. Arasaratnam-Smith

**Affiliations:** 70985 University of Algarve, Faro, Portugal; Centre for Research in Psychology (CIP/UAL) and 70985 University of Algarve, Faro, Portugal; Faculty of Business, Arts, Education and Social Sciences, 115530 Alphacrucis College, Sydney, Australia

**Keywords:** multicultural attitudes, perceived intergroup threat, attitudes toward immigrants, polynomial regression analysis, response surface methodology, *The world is less peaceful today than at any time in the last decade*

## Abstract

Supported by the Intergroup Threat Theory (ITT), this study analyzes the effect of multicultural ideology on attitudes towards immigrants mediated by realistic, symbolic, and zero-sum threats. With a sample of Portuguese participants (*N* = 404)), polynomial regression analysis with response surface methodology was used to test the effects of multicultural attitude (MA) and perceived intergroup threat (PIT) on attitudes towards immigrants (ATI). This study also tested a model in which positive MA leads to a lower PIT, and consequently to more favorable ATI. Four hypotheses were proposed; all of which were confirmed. The results further showed that the direction of discrepancy between MA and PIT can provide a more comprehensive understanding of the complex role of multicultural ideology in predicting ATI. Findings, limitations, and directions for future research are discussed.

## Introduction

Ethnic diversity in many societies is on the rise. According to a United Nations report on international immigration, 3% of the world’s population lives in a country other than its country of origin (United Nations, 2017). However, coexistence of different cultural groups has not always been peaceful and socially healthy. Data provided by the Global Peace Index (Institute for Economics & Peace, IEP, 2020) show that world peacefulness, defined as a complementarity between negative peace (absence of violence or fear of violence) and positive peace (attitudes, institutions and structures that create and sustain peaceful societies) has declined 2.5% since 2008. Europe, despite continuing to display the highest peace average, has witnessed a deterioration in the values of the three domains of the GPI, namely less safety security, more ongoing conflict and more militarization, for several years. Since 2008, 61% of European countries show a decrease in the peace index, including countries such as Iceland, Portugal, Austria, and Denmark, which are included in the group of the five most peaceful countries in the world (1st, 3rd, 4th and 5th place in the ranking of the GPI 2020, respectively). This decrease has been further accentuated in the last 5 years due to increase of internal conflicts and strained relations with neighboring countries. In 2020, there was a more pronounced effect on these indicators due to the insecurity and anxiety created by the COVID-19 pandemic and the consequent containment policies (IEP, 2020). These trends reflect a wide range of socially relevant events, such as terrorist attacks (e.g. New York, 2001; Madrid, 2004; London, 2005 and 2017; Paris, 2015; Barcelona, 2017, Nice 2015 and 2016) and economic and humanitarian crises that trigger or worsen tensions between nationals and immigrants (e.g., Allen & Nielsen, 2002; Bakhtiari, 2020; Buijs & Rath, 2002; Council on American-Islamic Relations, CAIR, 2007), causing racially motivated riots (e.g., Sidney, 2005), normalization of hate speech (Mulhall & Khan-Ruf, 2021), and the emergence of extreme right-wing policies and groups associated with negative attitudes towards minority groups (Barreto, 2018; Lange, 2020; PRC, 2019).

In Portugal, despite being a country with indicators favorable to multiculturalism in almost all indices (Migrant Integration Policy Index, MIPEX, 2020), the political party Chega, perceived as the extreme right, has been gaining voters in recent years. New nationalist extreme right groups have also emerged. The Defender Portugal Movement, for example, is identified in the European report on extremism in Europe (Mulhall & Khan-Ruf, 2021). The fall of the Berlin Wall in 1991 was a symbolic of marker of open borders and free movement in world history. However, in recent years there has been an increase in nationalist attitudes, xenophobia, (Kende & Krekó, 2020) and anti-immigration policies (e.g., Lange 2020).

Despite some countries’ efforts at parochialism, such as the construction of the United States-Mexico border wall, and despite growing desire for national isolationism in several countries (e.g., Gordon, 2017), social and cultural mobility remains a global reality.

While on the one hand increasing cultural diversity may be at the root of the aforementioned attitudes and trends, on the other hand, migration and global mobility contribute positively to economic growth (e.g., Dustmann & Frattini, 2014; Florida, 2002; Liu et al., 2020) and enhance quality of life for immigrants seeking new opportunities and better living conditions (e.g., Sandu et al., 2018).

That said, it must be noted that immigration does not always result in a better life (e.g., Borjas, 2003; Haddad & Balz, 2006; Rodrik, 1999). Immigrants are often exposed to hostile and discriminatory behaviors that negatively affect physical and psychological well-being (e.g., Bakhtiari, 2020; Berry & Hou, 2017; Gee 2002; Goosby et al., 2018; Pascoe & Richman, 2009; Samari et al., 2018). There is evidence of immigrants manifesting aggressive and counterproductive behaviors (e.g., Gürlek, 2021; Jiang & Chen, 2020), avoiding friendly relations with host nationals (Chen, 1999), and generally strained intercultural relations. The facilitation of better integration of immigrants into society benefits both immigrants and host nationals.

The development of indictors and measures that facilitate better policies and initiatives for social integration of ethnic minorities has been a focus of past research (Vijver et al., 2008). The success of integration facilitation initiatives depends on several conditions, not the least of which is the attitude of host nationals toward immigrants. Considering evidence of ambivalence of host national attitudes toward immigrants (e.g., Thompson et al., 1995) and the fact that ambivalent attitudes are less stable, (e.g., Bargh et al., 1992; Luttrell et al., 2016) and considering cultural variations in the predictors of attitudes toward immigrants, these variables warrant further scrutiny.

Inspired by the work of Ward and Masgoret (2006), the present study employs an integrative model of attitudes towards immigrants, supported by Stephan and Stephan’s Intergroup Threat Theory (1996, 2000) as a mediator of the effect of multicultural ideology on attitudes towards the immigrants. This research examines the effect of multicultural ideology on attitudes mediated by realistic threats and symbolic threats, as well as considering the perspective of zero-sum threats as proposed by Ward and Masgoret (2006).

The present study has two factors that distinguish it from others: firstly, it is empirically distinct in its use the attitudes recommended by the Eurobarometer. Secondly, unlike previous studies, the present study is carried out in the Portuguese context. As Portugal appears in several international instruments and indicators as demonstrating values that are mostly favorable to cultural diversity, the Portuguese context is pertinent for studying attitudes toward immigrants. Of further interest is that, Portugal, while demonstrating favorable values toward cultural diversity, does not score high in indicators of political participation and permanent residence (MIPEX) unlike Canada and New Zealand for example, where multiculturality seems more normative. Another distinctive of Portugal is the migratory flow in both directions. On the one hand, there is a significant increase in the emigration of highly specialized Portuguese young people in response to the crisis of the decade of 2010, while on the other hand immigration into Portugal from countries such as Brazil, Romania, and Ukraine has increased (SEF, 2019). Increased immigration flows are positively associated with perceived threats to national identity (e.g., Louis et al., 2013), and competition for resources (e.g., Esses et al., 2012), and support for nationalist groups.

## Multicultural Ideology and Attitudes toward Immigrants

Multicultural ideology, unlike other ideologies of diversity such as assimilation and colorblindness, specifically promotes diversity. Multicultural ideology values differences in memberships and identities– amongst people (Plaut, 2010; Rosenthal & Levy, 2010). Minimizing the differences between people to make them ‘the same’ is to disrespect individuals as unique and distinct identities. For example, while colorblindness is associated with a decrease in prejudice (e.g., Wolsko et al., 2000), it is also associated with less support for equality and integration policies (e.g., Yogeeswaran et al., 2018). Conversely, individuals who advocate multiculturalism consider that ethnic groups are not mutually exclusive and it is possible to live in harmony (Berry, 2006; Wolsko et al., 2006) while recognizing the distinctives unique to each ethnic group. Advocates of multiculturalism believe that the host society should create instruments and strategies, such as creating common group identity, that facilitate accommodating members of other cultural groups (Berry et al., 1977; Kunst et al., 2015). Multicultural attitudes encourage inclusive policies and behaviors, contributing to the reduction of prejudices and discriminatory behaviors (e.g., Richeson & Nussbaum, 2004a, 2004b; Wolsko, et al., 2006). In three studies comparing Spain and Canada, Urbiola et al. (2017) observed that multicultural ideology was negatively associated with prejudice and positively associated with social policies in support of minority groups (gypsies in Spain and First Nations people in Canada). However, the relationship between multicultural ideology and attitudes towards immigrants (and integration policies) is influenced by the perception of non-native groups as threatening (Sears, 1988; Tajfel & Turner, 1979). This explains why certain socio-economic events such as the impact of the COVID-19 pandemic on unemployment rates affect attitudes toward immigrants (e.g., Bakhtiari, 2020; Mulhall & Khan-Ruf, 2021). There is also evidence to suggest that attitudes toward multiculturalism varies amongst majority and minority members in a society, with majority members preferring assimilation of minorities, while minority members prefer to maintain their own cultural identity (Arasaratnam, 2013). These dynamics merit further investigation in the Portuguese context.

## Perceived Intergroup Threat and Attitudes toward Immigrants

According to Intergroup Threat Theory (ITT) there are four types of threats that negatively and concomitantly (or not) affect attitudes towards immigrants, namely, realistic threat, symbolic threat, negative stereotypes, and intergroup anxiety (Stephan et al., 1998). Realistic threats arise from scarcity of resources (employment opportunities, food, health, state support, etc.), which is why they are also called economic threats (e.g., Ha & Jang, 2015; Stephan et al., 2002). Symbolic threats constitute a threat to the worldview of the native society, to its norms, traditions, beliefs, and values. That is, symbolic threats constitute a threat to the national culture and identity (e.g., Ha & Jang, 2015; Stephan et al., 2002). Stereotypes serve as a basis for expectations about others, in this case, about immigrants in general and about each group of immigrants according to their country of origin, which in turn can lead to prejudice and discrimination (Stephan & Stephan, 1996). The fourth type of threat concerns intercultural interactions, the perception that natives can be rejected, ridiculed or exploited by immigrants (Arasaratnam, 2011). Several studies have observed the predictive effect of threats on attitudes towards immigrants. For example, a study analyzing the attitudes of Americans towards Mexicans and vice versa demonstrates the aforementioned four threats were predictors of attitudes in the two samples (Stephan et al., 2000).

Similar results were observed with American students in relation to immigrants from Cuba, Mexico, and Asia (Stephan et al., 1999) and Spanish and Israeli samples for Moroccan, Russian and Ethiopian immigrants (Stephan et al., 1998) and New Zealand samples for immigrants in general (Ward & Masgoret, 2006). Based on further research in which the results showed that threats mediate the impact of situational variables including intercultural distance on attitudes (contact, status, etc.), stereotypes were included as a distal variable in a review of the ITT model (Corenblum & Stephan, 2001; Stephan et al., 2002; Ward, & Masgoret, 2006). This review also contributed to several criticisms about the conceptualization of threats, negative stereotype, and intergroup anxiety, as well as antecedent factors that affect the degree of perceived threat that foreigners pose to the natives (Croucher, 2016; Riek et al., 2006; for a better understanding of the criticisms see Croucher, 2017). In the revised version of the ITT model, the authors introduce the concept of intergroup threat and identify two threats instead of the original four, namely realistic and symbolic threats (Stephan et al., 2009). Stereotype appears as a subset of both threats, the content of which concerns a risk to resources and security (realistic threat) or when the negative stereotype has the potential to damage the culture and identity of the native group (symbolic threat). Further, integroup anxiety is noted as a subset of realistic threats.

Studies conducted in the United States show that Latin immigrants are perceived as a realistic and symbolic threat to US Caucasian culture (e.g., Hall & Krysan, 2016). Pehrson and colleagues (2012) found that Irish Protestants and unionists (that is, those with historical power) were driven by cultural threats, thus predicting prejudice against ethnic minority groups and migrant workers. In other studies, in European countries, the results are similar (e.g., Croucher et al., 2014; Makashvili et al., 2018; Wirtz et al., 2016), although with some differences. In some studies, threats, when analyzed specifically, are not significant predictors of attitudes (e.g., Croucher, 2013; González et al., 2008). Other authors (e.g., Ward & Masgoret, 2006) propose the zero-sum belief as another type of threat. Zero-sum belief designates the general belief system about the antagonistic nature of social relations in which the gain of some is the loss of others. This system of beliefs contributes to the increase in the number and intensity of social conflicts, whether in societies with liberal or conservative ideologies, for which we consider the zero-sum threat as a threat in our study. Thus, considering three types of realistic, symbolic and zero-sum threat, we intend to test the effects of multicultural attitude (MA) and perception of intergroup threat (PIT) in a set of results related to attitudes towards immigrants (ATI) in the population Portuguese. We used polynomial regression analysis with response surface methodology.

Response surface analysis is a technique that provides a differentiated view of relationships between combinations of two predictor variables and a result variable, by graphically representing the results of polynomial regression analyzes in a three-dimensional space system (Shanock et al., 2010). This technique has more explanatory potential than difference scores or traditional moderate regression analyzes and has been used as a way to deal with the problems associated with the differences in scores and the differentiation between the degrees of discrepancy and agreement (e.g., Brunet et al., 2012). According to previous studies higher values of MA explain less perceived threat and, consequently, more favorable attitudes (e.g., Richeson & Nussbaum, 2004a, 2004b; Ward & Masgoret, 2006). Thus, based on such findings and previous research that used polynomial regression with response surface methodology (Cable & Edwards, 2004; Edwards & Cable, 2009; Shanock et al., 2010), we hypothesize that:

H1 There is a positive relationship between multicultural attitudes (MA) and favorable attitude toward immigrants (ATI) and a converse relationship between perceived intergroup threat (PIT) and favorable ATI.

Also, It is further expected that the direction of the discrepancy between the predictors of the study has an influence on ATI. That is, when the MA scores are higher compared to the PIT scores, favorable ATI scores will also be higher.

H2 Favorable ATI scores will be higher when MA is higher than PIT, and lower when MA is lower than PIT (direction of discrepancy hypothesis).

The degree of discrepancy between the predictors must also be considered.

H3 Higher positive discrepancy (i.e., MA is much greater than PIT) will be positively associated with favorable ATI, whereas higher negative discrepancy (i.e., MA is lower than PIT) will be positively associated with less favorable ATI (degree of discrepancy hypothesis).

Considering the multicultural ideology as an individual antecedent of attitudes towards immigrants (Berry et al., 1977), we further hypothesize that:

H4 The relationship between MA and ATI is mediated by PIT.

Finally, we intend to test a model in which positive multicultural attitudes lead to a lower perception of threat, and consequently to more favorable attitudes towards immigrants.

## Method

### Sample

Participants were native and permanent residents in Portugal and over 18 years of age (*N* = 404, m = 123, f = 281). Participants were aged between 18 and 82 years old (M = 40.94; SD = 15.43). The majority were married or living in common law (*n* = 196, 48.5%) and the rest were single (*n* = 162, 40.1%). The educational qualifications of the participants mainly correspond to secondary education (*n* = 178, 44.1%) and a degree level (*n* = 137, 33.9%). More than half of the participants identified as professionally active (*n* = 225, 55.7%).

### Instruments

In addition to a section on demographic information, the questionnaire included measurements of: Multicultural attitude, Perceived Intergroup Threat and Attitudes toward Immigrants*,* presented in that order.

All instruments were previously translated and tested for the Portuguese population. We applied the translation/back-translation procedure to translate English-based scales into Portuguese (Hambleton et al., 2005). The first step was the translation from English to Portuguese by two bilingual specialists working independently. These two versions were subsequently translated back into English by two other bilingual experts independently. The translations were compared to the original and adjusted by two psychologists specialized in the subject and native Portuguese. Inconsistencies of individual items due to translation problems were discussed and addressed, as well as the applicability to the Portuguese cultural context. To test the translation, 15 participants were asked to answer the Portuguese version (pre-test) to correct possible semantic problems, the usability and clarity of the items. No interpretation problems were detected. These participants were not included in the final sample.

*Multicultural Attitude Scale (MAS)* – based on the Multicultural Ideology Scale by Berry and Kalin (1995), it was adapted by Breugelmans and Van de Vijver (2004), with the aim of covering a broader range of aspects of multiculturalism, diversity, acculturation of minorities, support for minorities and equal rights and social participation. It is a one-dimensional scale composed of 19 items evaluated on a 7-point Likert scale (1 - I totally disagree to 7 - I totally agree). Examples of items: item 2 ”I think that the unity of Portugal is weakened by the presence of foreigners” and item 8: “I approve that foreign resident women wear headscarves.”).

*Perceived intergroup threat (PIT)* - the three scales developed by Ward and Masgoret (2006) were used to assess the feelings of threat and competition in relation to immigrants: realistic threat, symbolic threat (3 items each) and zero-sum beliefs (4 items). The original scale refers to New Zealand culture, so the items were adapted to the Portuguese reality and evaluated using a 7-point Likert scale (1-I totally disagree to 7 - I totally agree). Some examples of items are: “*How much do you agree or disagree that immigrants take jobs away from other Portuguese’s?” *(realistic threat); “*How much do you agree or disagree that immigration tends to threaten Portuguese culture?*” (symbolic threat); “*How much do you agree or disagree that the more political power immigrants obtain, the more difficult it is for Portuguese’s already living here?*’’ (zero-sum beliefs). Each dimension was added, and the mean was calculated to obtaining an overall score. Higher scores indicate stronger feelings of intergroup threat.

*Attitudes Towards Minority Groups (ATI) -* It is a scale developed by the Institute for Social Research and Analysis (SORA, 2001) (Institute for Social Research and Analysis SORA, 2001) and used in Eurobarometer surveys. This scale includes seven dimensions, which measure attitudes towards social minorities (the restrictive acceptance of immigrants’ dimension was not used in the present study). The remaining six dimensions were applied, namely: cultural assimilation (2 items, e.g., item 1: *To be fully accepted in Portugal, emigrants must abandon their own culture*)*;* blaming minorities (6 items, e.g., item 6: *Emigrants are often involved in crime)*; support for policies improving social coexistence (7 items, e.g. item1: *Penalizing discrimination against minority groups)*; disturbance (3 items, e.g., item 2: *Do you personally consider that the presence of people of another nationality disturbs your daily life?);* multicultural optimism (5 items, e.g., item 1: *Immigrants enrich Portuguese culture)* and conditions of repatriation (3 items, item 3*: Legally established emigrants who do not belong to the European Union must be sent back to the country of origin).* All scales are operationalized measures according to a Likert scale from 1 (strongly disagree) to 7 (strongly agree), except for the restrictive acceptance scale of the immigrant dimension, which uses five items of nominal response, which is why it was not used.

The means, standard deviations and internal consistency values of the variables are listed in Table 1.

**Table 1.** Means, Standard-Deviation, Internal Consistency and Correlations.

Variables	M	SD	1	2	3	4	5	6	7	8
1. MA	4.76	.84	(.846)							
2. PIT	2.93	1.18	−.680**	(.914)						
3. CA	2.77	1.32	−.270**	.289**	(.388)					
4. BLAM	3.15	1.23	−.557**	.792**	.306**	(.863)				
5. POL	5.25	1.08	.568**	−.344**	−.109*	−.279**	(.854)			
6. DIST	1.81	1.12	−.510**	.507**	.301**	.460**	−.351**	(.911)		
7. MO	4.98	1.25	.669**	−.476**	−.170**	−.361**	.580**	−.387**	(.919)	
8. CR	2.43	1.33	−.523**	.592**	.159**	.511**	−.292**	.380**	−.364**	(.858)

Legend: MA – Multicultural attitude; PIT – Perceived intergroup threat; CA – Cultural assimilation; BLAM – Blaming minorities; POL – Policies improving social coexistence; DIST – Disturbance; MO – Multicultural optimism; CR – Conditional repatriation; Note: Cronbach’s alpha in brackets; **p ≤ .01; *p ≤ .05.

### Procedures

#### Data Collection

Data were collected by means of a self-reported questionnaire on paper, face-to-face (average completion time 10 minutes). Participation was voluntary and unpaid, and participants were guaranteed the rights to freedom of participation, anonymity, and data confidentiality. The questionnaires were collected by two researchers of the same cultural ethnicity as the respondents to minimize the possibility of socially desirable answers. The context for data collection varied from university classes to workplaces and homes of participants. The study followed human research ethics criteria approved by the Center’s Ethics Committee of the research center to which two authors belong.

### Data analysis

**Figure 1.**
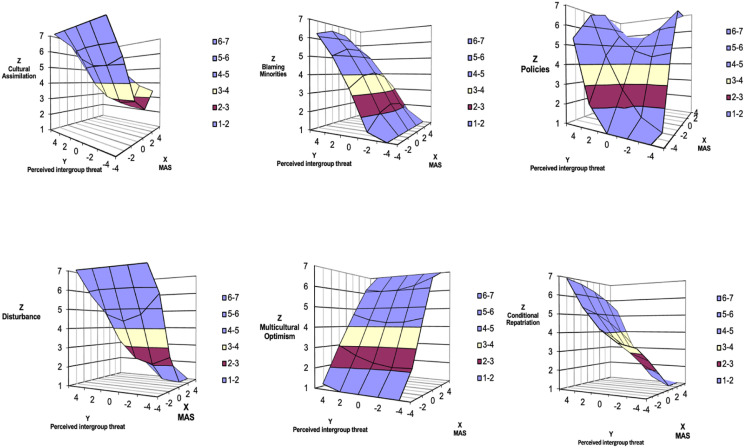
Three-dimensional representation of the combined effect of the multicultural attitude and perceived intergroup threat on the attitudes towards immigrants: Response surface analysis graphs.

Data were analyzed with SPSS software (v.26) using polynomial regression analyses with response surface methodology. In this methodology, polynomial regression is performed first, using the formula: Z = b_0_ + b_1_X + b_2_Y + b_3_X^2^ + b_4_XY + b_5_Y^2^ + *e*, where Z is a dependent variable, X is Predictor 1 (MA), and Y is Predictor 2 (PIT) (Shanock et al., 2010). Thus, MA and PIT were centered and modeled as separate predictors (X_1_ and Y_2_) along with the square of these centered variables (X_1_^2^ and Y_2_^2^) and the cross product of these centered variables (X_1_ x Y_2_) to assess the linear, nonlinear, and interactive relationships between the MA and PIT and each outcome of attitudes toward immigrants. Next, the regression coefficients were transformed into four surface values (a1 to a4). These values were used to examine how the degree of agreement/discrepancy and the direction of the discrepancy between MA and PIT related to each outcome. Then, a three-dimensional graph was drawn up that corresponds to the combinations of the regression coefficients and allows the interpretation of the values from a1 to a4 (Edwards, 1994; Shanock et al., 2010). Results of the polynomial regression analyses and response surface methodology are presented in Table 2 and Figure 1.

**Table 2.** Polynomial Regression and Response Surface Analysis.

Variable	CA	BLAM	POL	DIST	MO	CR
Polynomial regression coefficients						
*β0*	3.120 (.100)***	4.089 (.059)***	4.740 (.068)***	2.354 (.073)***	4.216 (.076)***	3.196 (.084)***
*β1*	−.521 (.151)***	−.029 (.090)	.716 (.103)***	−.602 (.111)***	.941 (.118)***	−.314 (.127)**
*β2*	.260 (.100)**	.766 (.059)***	.301 (.068)***	.310 (.073)***	.028 (.076)	.566 (.084)***
*β3*	.061 (.107)	−.053 (.064)	−.216 (.073)**	.151 (.079)	−.042 (.091)	.031 (.090)
*β4*	.220 (.106)*	−.035 (.063)	−.143 (.072)*	.207 (.078)**	−.019 (.082)	−.016 (.090)
*β5*	.055 (.051)	−.050 (.031)	.065 (.035)	.086 (.038)*	.021 (.044)	.055 (.044)
*R*^*2*^	.117	.631	.375	.334	.452	.379
Surface test						
*a1*	−.26 (.21)	.74 (.12)***	1.02 (.15)***	−.29 (.15)	.97 (.17)***	.25 (.18)
*a2*	.34 (.22)	−.14 (.13)	−.29 (.15)*	.44 (.16)**	−.04 (.19)	.07 (.18)
*a3*	−.78 (.14)***	−.80 (.10)***	.42 (.10)***	−.91 (.11)***	.91 (.11)***	−.88 (.12)***
*a4*	.21 (.07)**	−.07 (.03)*	.14 (.04)***	.14 (.04)***	.04 (.05)	.01 (.06)

Legend: CA – Cultural assimilation; BLAM – Blaming minorities; POL – Policies improving social coexistence; DIST – Disturbance; MO – Multicultural optimism; CR – Conditional repatriation. *Note.* *p <.05, **p <.01, and ***p <.001 denote the level of significance.

The mediation analysis was performed using the Macro Process for SPSS, using the simple mediation model four proposed by Hayes (2018). The research model was tested using the structural equation modeling (SEM) in SPSS AMOS and the Maximum Likelihood Estimation method were used. The following goodness of fit indicators were calculated based on the recommendations of Byrne (2001): (1) χ2, a significance test of the minimized discrepancy function during model fitting (the lower the value, the better the adjustment; Marôco 2014); (2) CMIN/DF, which corresponds to the data adjustment probability to the theoretical model (values must vary between 2 and 5); (3) the Comparative Fit Index (CFI), Normed Fit Index (NFI) and Tucker-Lewis Index (TLI), which vary between 0 and 1 (values >.90 indicate good fit; Bentler & Bonett, 1980); and (4) the Root-Mean Square Error of Approximation (RMSEA)—an ideal value is between.05 and.08, with values up to.10 considered acceptable (e.g., Browne & Cudeck, 1993).

## Results

### Descriptive Statistic

As noted in Table 1, multicultural attitudes (MA) has a mean of 4.76 (SD = .84) and perceived intergroup threat (PIT) has a mean of 2.93 (SD = 1.18). Within the variables of attitudes toward immigrants (ATI), support for policies improving social coexistence had the highest mean (M = 5.25, SD = 1.08) and disturbance the lowest mean (M = 1.81, SD = 1.12). It can be concluded that, in general, the variables associated with negative ATI have lower means, compared to the variables associated with favorable attitudes. All variables correlated in a statistically significant way (*p* < .05). MA was negatively correlated with PIT and with all variables associated with less favorable ATI: cultural assimilation, blaming minorities, conditions of repatriation and disturbance. On the other hand, PIT was negatively correlated with variables related to favorable ATI: multicultural optimism and policies improving social coexistence.

### Polynomial Regression with Response Surface Analysis

Results of the polynomial regression analyses and response surface methodology are presented in Table 2 and figure 1. The regression model is significant for cultural assimilation (*F* (5,388) = 10.302, *p* < .001, *R*^2^ = .117). MA and PIT both negatively and positively predict cultural assimilation, respectively. There was no significant linear (a1 = −.26, SE = .21, *p* = .218) or quadratic (a2 = .34, SE = .22, *p* = .131) effect of MA and PIT on cultural assimilation along the congruence line. The cultural assimilation is lower when the MA is higher than the PIT (a3). A positive and significant a4 reveals that the further MA and PIT deviate from each other, the higher the cultural assimilation.

The regression model for blaming minorities is also statistically significant (*F* (5,388) = 131.944*, p* < .001, *R*^*2*^ = 631) and shows that perceived intergroup threat (PIT) has a positive predictive effect on blaming minorities. The degree of agreement between multicultural attitude (MA) and PIT was linearly and positively associated with blaming minorities (a1). That is, blaming minorities is higher when MA and PIT combine at higher levels than at lower levels. When MA has a higher value compared to PIT, the blaming minorities value is lower (a3).

The regression model for support for policies improving social coexistence (POL) was also significant (*F* (5,384) = 45.998, *p* < .001, R2 = .375). There was a significant linear (a1 = 1.02, SE = .15, *p* < .000) and quadratic (a2 = −.29, SE = .15, *p* = .047) effect of MA and PIT on POL, along the congruence line. As indicated by a positive a3 effect, the direction of the discrepancy has an effect on POL. That is, higher values on the POL are observed when the MA is higher compared to the PIT. A positive and significant a4 reveals that the more MA and PIT deviate from each other, higher the support for POL.

Regarding the disturbance variable, both MA and PIT are negative and positive predictors, respectively (*F* (5,389) = 38.960, *p* < .001, *R*^*2*^ = .334). A positive and significant a2 effect indicates that disturbance is higher when MA and PIT combine at more extreme levels than at midrange levels. The disturbance is lower when the MA is higher than the PIT (a3). A positive and significant a4 reveals that the more MA and PIT deviate from each other, the higher the disturbance.

The regression model for multicultural optimism (MO) is also statistically significant (*F* (5,385) = 63.554, *p* < .001, *R*^*2*^ = .452), observing that MA is a strong predictor of this variable. The degree of agreement between MA and PIT was linearly and positively associated with MO (a1). Along the incongruence line, the a4 effect was not significant; the degree of discrepancy has no impact on the MO. However, as indicated by a positive a3 effect, the direction of the discrepancy influences the MO. That is, higher values on the MO are observed when the MA is higher compared to the PIT (a3).

Finally, the regression model for conditions of repatriation (CR) is a statistically significant model (*F* (3,387) = 47.165, *p* < .001, *R*^*2*^ = .379), observing that MA is a negative predictor and PIT a positive predictor. There was no significant linear (a1 = −.26, SE = .21, *p* = .218) or quadratic (a2 = .34, SE = .22, *p* = .131) effect of MA and PIT on CR over congruence line. A significant negative a3 indicates that CR is higher when PIT is higher than MA. Along the incongruence line, the a4 effect was not significant, that is, the degree of discrepancy has no impact on the CR.

### Mediation Analysis

The results of the mediation analysis can be seen in Table 3. Perceived intergroup threat (PIT) proved to be a mediator of the relationship between multicultural attitudes (MA) and the variables related to less favorable attitudes towards immigrants. For cultural assimilation, the indirect effect was *ß* = −.20 (95% BCa CI = −.3672 – -.0491). For blaming minorities, the mediation effect (indirect effect) of PIT was significant (*ß* = −.76, 95% BCa CI = −.8919 – -.6591). PIT also showed an indirect effect on the disturbance variables (*ß* = −.26, 95% BCa CI = −.4083 – -.1329) and CR (*ß* = −.48, 95% BCa CI = −.6626 – -.3168). For POL and multicultural optimism, the indirect effects were not statistically significant, because zero was contained in the confidence interval bootstrap.

**Table 3.** Model Coefficients for the Attitudes Toward Immigrants (Mediation Analysis).

Antecedent		Consequent						
		MA (PIT)	CA	BLAM	POL	DIST	MO	CR
		Coef (ep)	Coef (ep)	Coef (ep)	Coef (ep)	Coef (ep)	Coef (ep)	Coef (ep)
MA PIT	a	−.953 (.25)*** R^2^ = .463 F (1,392) = 336.89, p < .001	c` −.233 (.10)* b .214 (.07)* R^2^ = .095 F (2,391) = 20.55, p < .001					
MA PIT	a	−.956 (.05)*** R^2^ = .463 F (1,390) = 336.24, p < .001		c` −.044 (.06) b .804 (.04)*** R^2^ = .63 F (2,389) = 328.27, p < .001				
MA PIT	a	−.956 (.05)*** R^2^ = .464 F (1,388) = 335.57, p < .001			c` .801 (.07)*** b .075 (.05) R^2^ = .328 F (2,387) = 94.36, p < .001			
MA PIT	a	−.955 (.05)*** R^2^ = .462 F (1,393) = 338.69, p < .001				c` −.427 (.07)*** b .277 (.05)*** R^2^ = .31 F (2,392) = 88.61, p < .001		
MA PIT	a	−.963 (.05)*** R^2^ = .476 F (1,389) = 353.50, p < .001					c` .977 (.07)*** b −.027 (.05) R^2^ = .449 F (2,388) = 158.62, p < .001	
MA PIT	a	−.950 (.05)*** R^2^ = .461 F (1,391) = 334.49, p < .001						c` −.344 (.08)*** b .509 (.06)*** R^2^ = .375 F (2,390) = 117.44, p < .001

Legend: MA – Multicultural attitude; PIT – Perceived Intergroup threat; CA – Cultural assimilation; BLAM – Blaming minorities; POL – Policies improving social coexistence; DIST – Disturbance; MO – Multicultural optimism; CR – Conditional repatriation. *Note.* *p <.05, **p <.01, and ***p <.001 denote the level of significance.

### Structural equation model

**Figure 2.**
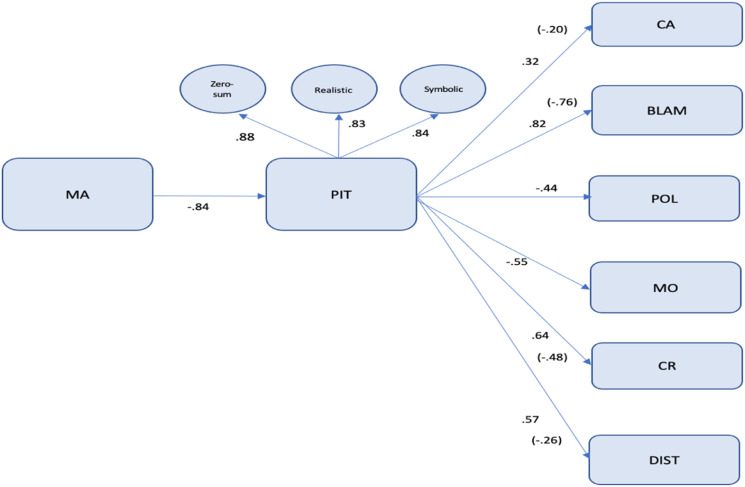
Structural equation model.

Legend: MA – Multicultural attitude; PIT – Perceived Intergroup threat; CA – Cultural assimilation; BLAM – Blaming minorities; POL – Policies improving social coexistence; DIST – Disturbance; MO – Multicultural optimism; CR – Conditional repatriation. (*n* = 404; standardized coefficients; indirect effects in brackets).

Figure 2 shows results of the path analysis performed on a hypothesized model. The model was tested across the sample (*n* = 404). The resulting χ2 is 287.917 with 33 degree of freedom (*p* = .000); CMIN/DF = 8.72; RMSEA = .13; NFI = .87; CFI = .88; TLI = .81. The values are all close to the acceptable recommended value of .90, suggesting that the model provides a robust representation of the relationships among the variables in the proposed model. Only the RMSEA value is above the considered as acceptable. While the Multicultural attitudes (MA) represents an exogenous latent variable, the remaining seven latent constructs in the model are designated as endogenous variables, i.e., variables that are influenced by (and may influence) other latent variables. The endogenous variables are: Perceived intergroup threat (PIT), cultural assimilation (CA), blaming minorities (BLAM), policies improving social coexistence (POL), multicultural optimism (MO), conditional repatriation (CR) and disturbance (DIST). Based on these relationships in the model, perceived intergroup threat relates directly to positive and negative attitudes toward immigrants.

## Discussion

This study aimed to test the effects of multicultural attitude (MA) and perceived intergroup threat (PIT) on attitudes towards immigrants (ATI), using polynomial regression with response surface methodology. Polynomial regression analysis with response surface methodology has similarities with traditional regression analyses because it retains the ability to examine the unique associations of MA and PIT with specific results.

Our results showed that MA is a consistent predictor of favorable ATI showing moderate, positive and statistically significant correlation values with support for policies improving social coexistence and multicultural optimism. In contrast, PIT was a more consistent predictor of results with negative valences (cultural assimilation, blaming minorities, disturbance and conditions of repatriation), presenting negative correlations with favorable ATI and positive correlations with unfavorable ATI. That is, as expected, higher values of multicultural attitudes explain less perceived threat and, consequently, more favorable attitudes toward immigrants, thus supporting H1.

H2 was also supported by the fact that the direction of the discrepancy between the MA and PIT showed positive effects on the variables policies improving social coexistence (POL) and multicultural optimism (MO). That is, when the MA is higher compared to the PIT, the result variables showed higher values. On the contrary, when the direction of the discrepancy between the two predictors had negative and statistically significant effects on the variables cultural assimilation (CA), blaming minorities (BLAM), disturbance (DIST) and conditional repatriation (CR), they showed lower values when the PIT is less than the MA. This therefore supported H2 which hypothesized that favorable ATI have higher scores when MA is higher compared to PIT, and less favorable ATI have higher scores when MA is lower than PIT.

As for H3, the results showed higher scores in CA, POL, and DIST with increased deviation in MA and PIT scores. On the contrary, with regard to the BLAM variable, a significant negative a4 was observed, which shows the existence of a degree of negative discrepancy between the predictors, in this case, lower MA values are associated with lower BLAM scores. The degree of discrepancy between MA and PIT therefore has an impact on the observed results, thereby supporting H3.

The mediation analysis showed that PIT is a mediator of the relationship between MA and ATI, thus confirming H4. The direct effect of MA on ATI is reduced with the entry of the PIT mediator, except for the BLAM variable, whose value increased with the addition of the mediator, since the direct effect of MA was not statistically significant. It should also be noted that in the most favorable ATI, i.e., POL and MO, the predictive value of PIT (*b*) was not statistically significant (*p* > .05).

The objective of this investigation was also to test a model in which positive multicultural attitudes lead to a lower perception of threat, and consequently to more favorable attitudes towards immigrants. The results of the model showed that MA is a negative predictor of PIT; that is, positive multicultural attitudes reduce the perception of threat. On the other hand, PIT proved to be a negative predictor of favorable attitudes towards immigrants, in this case, of POL and MO variables. As postulated by Ward and Masgoret (2006), we positioned MA as an exogenous variable and the rest as endogenous variables. The model demonstrated good adjustment indexes with the exception of the RMSEA, which, according to (Kenny et al., 2015), may be associated with a recurrent sampling error in models with low degrees of freedom and small samples which can give rise to values artificially high levels of RMSEA.

In sum, the results of the present study support findings in previous research. Specifically, the results show that the direction of discrepancy between multicultural attitude (MA) and perceived intergroup threat (PIT) can provide a more comprehensive understanding of the complex role of multicultural ideology in predicting attitudes towards immigrants. In fact, in all six dimensions, surface values revealed that higher MA scores than PIT were related to more favorable attitudes toward immigrants (ATI), while higher PIT scores compared to MA reflect less favorable ATI.

According to our results, PIT has a greater effect on ATI than MA. On the one hand, these results show that MA is not sufficient to guarantee the stability of favorable ATI and, on the other hand, ATI are negatively affected by certain events or even prejudiced attitudes. Conceptually, attitudes are considered relatively stable (e.g., Eagly & Chaiken, 1993, Petty et al., 2007), allowing to predicted future behavior. However, the stability of attitudes is associated with strong attitudes, i.e., attitudes with a high degree of certainty and resistant to change (e.g., Krosnick & Petty, 1995; Luttrell et al., 2016). When attitudes are weak or ambivalent, they are attitudes that change more easily through persuasive speeches or direct experiences (Luttrell et al., 2016). The same applies to ATI, which, being ambivalent, are associated with systematic processing (Maio et al., 1996), but with slow evaluations and low attitude stability (e.g., Bargh et al., 1992; Liver et al., 2007) and hence more susceptible to change. The processing model recommended by Petty and Cacioppo (1986) states that favorable attitudes result from peripheral and not central processing and are more easily alterable, particularly if they are weak attitudes (Petty & Cacioppo, 1986). Several studies have shown that negative emotions imply a higher level of processing than neutral or weakly positive emotions (e.g., Moons & Mackie, 2007; Petty & Briñol, 2015; Stavraki et al., 2021) contributing to the strength and stability of negative attitudes compared to positive attitudes. It is therefore reasonable to observe that, in relation to immigrants, negative events (e.g., competition for jobs, social conflicts, terrorism), hate speech and/or prejudice levels stimulate more prominent negative emotions than positive events contributing to unfavorable ATI and multicultural policies (e.g., Matthes et al., 2019).

The ambiguity characteristic of the Portuguese ATI may explain the fact that the Portuguese have excellent results in the various indicators and rankings considered (e.g., GPI; MIPEX), but not be characterized by a normative multiculturalism as evidenced in other countries (e.g., Canada, New Zealand). It should therefore be noted that the indicators of Portugal that presented lower values refer precisely to the issues of integration of immigrants in a definitive way (i.e. political participation and permanent residence, MIPEX). Thus, the acceptance of immigrants appears “conditional” and subject to the immigrant having “foreigner” status. According to the “population change paradigm”, several studies have shown that the increase in the immigrant population causes an increase in perceived threat among the host community (e.g., Craig & Richeson, 2017). The results in these indicators thus express one of the ways of the Portuguese population to respond to the increase in immigration (SEF, 2019) trying to control the increase in minority groups.

### Limitations and Implications

The problem of multicultural interactions and harmonious cultural diversity is extremely complex and encompasses a wide range of constructs and processes. As such, despite the robustness of our model and the techniques of statistical analysis, the present study is not without its limitations. In addition to the unsatisfactory value of RMSEA, the low alpha value of the cultural variable assimilation is also noteworthy. The high RMSEA value can be associated with the size of the sample or the size of the model in relation to the data. The low alpha value could be due to a low number of questions (in this case, 2 items), or heterogeneous constructs (Tavakol & Dennick, 2011). Future studies should consider this limitation. The instrument used, besides being self-reported, always considered the term “immigrant” or “foreign”, without allowing for nuances of cultural distance, reasons for migration, and other related variables. Although several studies explore this measure, we note that the items used to measure perceived threats can also be used to measure prejudice towards immigrants. That is, the level of agreement for these items may indicate the participants' levels of prejudice rather than the level of threat perception. Future research should employ complementary methods to examine attitudes towards immigrants as well as biographical and cultural distance characteristics.

Contrary to other studies, the weight of realistic, zero-sum and symbolic threats at PIT is very similar in the present findings. We believe that it is realistic threats that suffer the most from negative events, negatively affecting attitudes toward immigrants – a belief that needs to be tested in future studies. Given the number of immigrants and support for extreme right movements has increased significantly in Portugal, it is essential to analyze the extent to which these variables contribute to a collective existential threat in Portugal, as reported by several studies with other populations (e.g., Bai & Federico, 2021).

Finally, future studies should examine the effect of emotions triggered by negative events on the perception of threat and on the formation and stability of favorable attitudes toward immigrants. A better understanding of the relationship between these variables will facilitate more effective and lasting strategies and policies for normative multiculturalism.
